# Conductivity Tensor Imaging of the Human Brain Using Water Mapping Techniques

**DOI:** 10.3389/fnins.2021.694645

**Published:** 2021-07-30

**Authors:** Marco Marino, Lucilio Cordero-Grande, Dante Mantini, Giulio Ferrazzi

**Affiliations:** ^1^Research Center for Motor Control and Neuroplasticity, KU Leuven, Leuven, Belgium; ^2^IRCCS San Camillo Hospital, Venice, Italy; ^3^Biomedical Image Technologies, ETSI Telecomunicación, Universidad Politécnica de Madrid and CIBER-BBN, Madrid, Spain

**Keywords:** conductivity tensor imaging, magnetic resonance imaging, electrical properties tomography, water content mapping, diffusion tensor imaging

## Abstract

Conductivity tensor imaging (CTI) has been recently proposed to map the conductivity tensor in 3D using magnetic resonance imaging (MRI) at the frequency range of the brain at rest, i.e., low-frequencies. Conventional CTI mapping methods process the trans-receiver phase of the MRI signal using the MR electric properties tomography (MR-EPT) technique, which in turn involves the application of the Laplace operator. This results in CTI maps with a low signal-to-noise ratio (SNR), artifacts at tissue boundaries and a limited spatial resolution. In order to improve on these aspects, a methodology independent from the MR-EPT method is proposed. This relies on the strong assumption for which electrical conductivity is univocally pre-determined by water concentration. In particular, CTI maps are calculated by combining high-frequency conductivity derived from water maps and multi b-value diffusion tensor imaging (DTI) data. Following the implementation of a pipeline to optimize the pre-processing of diffusion data and the fitting routine of a multi-compartment diffusivity model, reconstructed conductivity images were evaluated in terms of the achieved spatial resolution in five healthy subjects scanned at rest. We found that the pre-processing of diffusion data and the optimization of the fitting procedure improve the quality of conductivity maps. We achieve reproducible measurements across healthy participants and, in particular, we report conductivity values across subjects of 0.55 ± 0.01$\frac{S}{\text{m}}$, 0.3 ± 0.01$\frac{S}{\text{m}}$ and 2.15 ± 0.02$\frac{S}{\text{m}}$ for gray matter (GM), white matter (WM), and cerebrospinal fluid (CSF), respectively. By attaining an actual spatial resolution of the conductivity tensor close to 1 mm in-plane isotropic, partial volume effects are reduced leading to good discrimination of tissues with similar conductivity values, such as GM and WM. The application of the proposed framework may contribute to a better definition of the head tissue compartments in electroencephalograpy/magnetoencephalography (EEG/MEG) source imaging and be used as biomarker for assessing conductivity changes in pathological conditions, such as stroke and brain tumors.

## Introduction

The brain is a conductive medium characterized by different electrical conductivity values within each tissue type. Electrical conductivity defines how electrical currents propagate in biological tissues, and it depends on the tissue specific structures and composition (McCann et al., 2019), which might be altered in presence of pathological conditions, such as stroke and tumors (Katscher et al., 2013; Shin et al., 2015; Balidemaj et al., 2016a,b; Jensen-Kondering et al., 2020). Thus, being able to measure electrical conductivity in the brain *in vivo* is of great interest.

Electrical impedance tomography (EIT) measures electrical conductivity by injecting low frequency (LF) currents through electrodes placed on the scalp. With this technique, surface voltages are measured and then processed to reconstruct the conductivity values of the brain *via* the inversion of the Laplace equation. EIT techniques are subject to limitations, including: (i) the need to place electrodes around the head, (ii) the ill-posed nature of the inverse solution of the Laplace equation (Sylvester and Uhlmann, 1987; Chauhan et al., 2017), and (iii) the presence of the skull that by having high impedance values tends to divert the current away, which has a negative impact on the sensitivity of the technique itself (Oh et al., 2009; Chauhan et al., 2017). EIT techniques have been combined with magnetic resonance (MR) imaging (MR-EIT) to improve the spatial resolution of the conductivity maps. This is achieved by processing the information enclosed within the MR signal (Oh et al., 2003; Seo and Woo, 2014). Recently, MR-EIT has been combined with diffusion tensor imaging (DTI) data (Jeong et al., 2016; Chauhan et al., 2017) to probe the anisotropy of the conductivity tensor, which is prominent at the physiological LF range of the brain at rest, i.e., approximately 10 Hz.

MR electric properties tomography (MR-EPT) measures conductivity values using conventional MR sequences without the need of injecting currents in the head (Voigt et al., 2011). In MR-EPT, the conductivity is assumed to be embedded within the trans-receiving phase of the MR signal, which can be estimated *via* the Helmholtz equation (Borsic et al., 2015; Leijsen et al., 2019). However, the conductivity values are relative to high frequencies (HF), i.e., the resonance frequency of the MR scanner, or Larmor frequency—which for 3T systems corresponds to 128 MHz. While determining HF conductivity values allows, for example, a more accurate calculation of the specific absorption rate (SAR) (Katscher et al., 2009), it does not measure the electrical properties within the frequency range of the brain at rest. Furthermore, MR-EPT methods do not capture the anisotropic nature of the conductivity tensor of the brain at LF (Katscher et al., 2013), which is especially relevant for white matter (WM) fiber tracks, where conductivity values are higher along the main direction of the fibers.

Similarly to MR-EIT (Jeong et al., 2016; Chauhan et al., 2017), a recent study (Sajib et al., 2018) proposed an MR-EPT based technique to probe the anisotropy of the conductivity tensor. The conductivity tensor imaging (CTI) method combines MR-EPT measurements with multi b-value DTI data, exploiting the correlation that exists between water diffusivity and electrical conductivity (Sen and Torquato, 1989; Tuch et al., 2001; Sajib et al., 2018). Since their introduction, CTI methods (Sajib et al., 2018; Jahng et al., 2020; Lee et al., 2020) enabled measuring the conductivity tensor at LF using conventional MR scanners, open source software (Sajib et al., 2017), and without requiring extra hardware.

MR-EPT techniques are based on the processing of the phase of the MR signal. However, there are limitations including: (i) the assumption of spatial symmetry of the electric media when halving the trans-receiver phase (Van Lier et al., 2012), (ii) the involvement of the Laplace operator when solving the Helmholtz equation, which is sensitive to noise (Ropella and Noll, 2017), and (iii) the challenges posed by tissue boundaries with different conductivity values, where a reliable estimate of the Laplace is hard to obtain without substantial pre-smoothing and/or regularization (Katscher et al., 2013; Ropella and Noll, 2017).

Previous studies have suggested an association between the electrical properties of the tissues and their water concentration (Schepps and Foster, 1980). For example, a decrease in water content due to aging has been associated with decreased conductivity measurements (Peyman et al., 2001). Building on these observations, a recent study proposed a method to achieve HF isotropic electrical conductivity mapping (Michel et al., 2017). More specifically, by considering that the water in biological tissues [rather than electrolyte concentrations (Choi et al., 2020)] is a strong predictor of tissue’s electrical properties at HF, a direct relationship between water content and conductivity maps was built. Crucially, since the conductivity values were obtained without relying explicitly on the Helmholtz equation and thus on the Laplace operator, this method is by construction less sensitive to artifacts commonly found in MR-EPT. Consequently, despite this strong assumption, water mapping techniques might represent an alternative approach for CTI.

In this study, we propose a framework to achieve CTI in the human brain using water mapping techniques. Our methodology seeks to overcome the limitations of previous approaches, such as CTI mapping based on MR-EPT, by designing a framework independent from the Laplace operator. This objective is achieved by combining HF conductivity mapping based on water maps (Michel et al., 2017) and multi b-value DTI data (Tuch et al., 2001; Sajib et al., 2018; Jahng et al., 2020; Lee et al., 2020). Care is devoted toward a pipeline that optimizes (i) the acquisition of the DTI data, which is accelerated by means of multiband (Larkman et al., 2001) and SENSE (Pruessmann et al., 1999), (ii) the DTI pre-processing—for which a dedicated framework is employed to reduce noise and to correct for susceptibility induced spatial distortions and motion—and (iii) the fitting of a multi-compartment neurite orientation dispersion and density imaging (NODDI) (Zhang et al., 2012)—like model used to calculate the conductivity tensor (Lee et al., 2020). We tested our methodology on five healthy volunteers scanned at rest. Our results suggest that it is possible to attain high-signal-to-noise ratio (SNR) CTI maps at approximately 1 mm in-plane isotropic resolution without substantial image blurring/artifacts at tissue boundaries.

## Materials and Methods

### Overview

The CTI framework relates the conductivity tensor **C**_*LF*_ at LF to the extracellular water diffusion tensor **D**_*e*_ (Sajib et al., 2018):

(1) $${\text{C}}_{L\,F}=\frac{\chi_{e}\,\sigma_{H\,F}}{\chi_{e}\,d_{e}+\left(1-\chi_{e}\right)\,d_{i}\,\beta }\,{\text{D}}_{e}$$

In this equation, σ_*HF*_ represents the isotropic conductivity at HF, χ_*e*_ the extracellular volume fraction, *d_i_* and *d_e_* intra and extracellular water diffusivities, respectively, and β=0.41 the ratio of ion concentrations between intra and extracellular spaces. Note that the value for β was retrieved from a recent publication (Sajib et al., 2018). Equation 1 assumes a linear relationship between the electrical conductivity tensor and the extracellular diffusion tensor. Different processing pipelines have been developed for the estimation of σ_HF_, χ_e_, *d_i_*, *d_e_* and **D**_*e*_ (Sajib et al., 2018; Jahng et al., 2020; Lee et al., 2020).

**FIGURE 1:**
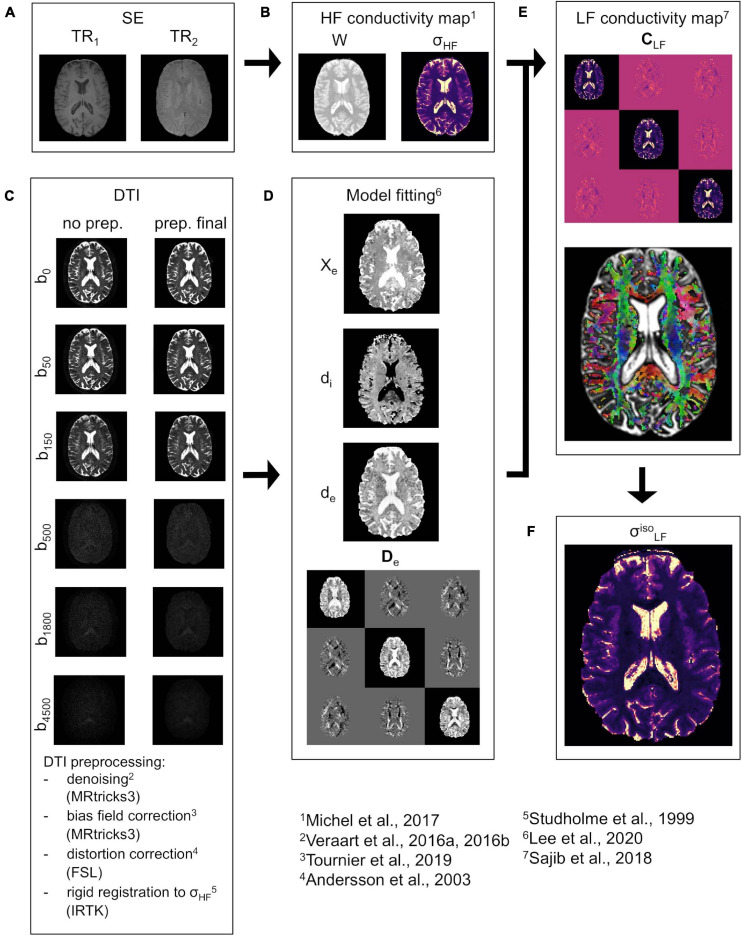
CTI reconstruction framework. **(A)** SE data acquired at TR_1_ and TR_2_. **(B)** Water map *W* and isotropic conductivity σ_*HF*_ map. **(C)** DTI data before (left) and after (right) pre-processing at all b-values (vertical direction, diffusion encoding direction chosen at random). **(D)** Estimated extracellular volume fraction χ_*e*_, intra and extracellular water diffusivities *d_i_* and *d_e_*, and extracellular water diffusion tensor **D**_*e*_. **(E)** LF conductivity tensor **C**_*LF*_ (top) together with the orientation distribution function (ODF) representation (bottom) in WM and GM. **(F)** “isotropic equivalent” LF conductivity map $\sigma_{L\,F}^{i\,s\,o}$.

In the following, a brief overview of the proposed pipeline is provided. The pipeline uses an MR-based framework, which combines spin echo (SE) images and multi b-value DTI data (Figure 1). The whole procedure is oriented toward the reconstruction of the conductivity tensor **C**_*LF*_.

For the estimation of σ_*HF*_, two SE images at different repetitions times (TR_1_ and TR_2_, Figure 1A) are acquired. These are then fused to compute a water map *W* (Figure 1B) which was employed to derive σ_*HF*_ (Michel et al., 2017). The procedure is explained in detail in section “HF Conductivity Estimation.” Concerning the DTI, the data is pre-processed according to an optimized pipeline (Figure 1C), which is described in section “DTI Pre-processing.” The pre-processed DTI data is used to derive **D**_*e*_ and to estimate *d_i_*, *d_e_*, and χ_*e*_ (Figure 1D) using a recently proposed model (Lee et al., 2020). A sub-routine that optimizes the fit for *d_i_*, *d_e_*, and χ_*e*_ is also proposed in section “Model Fitting”. All data is compounded to reconstruct **C**_*LF*_ by means of Equation 1 (Figure 1E). To validate the proposed methodology, an “isotropic equivalent” LF conductivity map σ${}_{L\,F}^{i\,s\,o}$ (Figure 1F) is constructed from **C**_*LF*_.

The source code, together with a sample dataset and relevant documentation specifying software requirements etc., is freely available at https://github.com/gferrazzi/CTI_mapping.

### Image Acquisition

The methodology has been validated on five healthy volunteers (S1 to S5, one female, mean age 38 ± 5 years) scanned on a 3T Philips Ingenia scanner equipped with a 32-channel receiver head coil. This study was approved by the regional scientific ethics committee and written informed consent was obtained from all participants.

The acquisition protocol consists of the following:

#### Anatomical Scan

A 3D magnetization prepared rapid acquisition gradient echo (MPRAGE) scan was acquired at 1 mm isotropic resolution, field of view (FOV) = 256 × 240 × 240 mm^3^, repetition/echo time TR/TE = 8.1/3.7 ms, inversion time TI = 950 ms, SENSE acceleration 2 and 2.6 along primary [Right-Left, (RL)] and secondary [Foot-Head, (FH)] phase encoding (PA) directions, flip angle (FA) = 8°. Total scan time was 5 min and 13 s.

#### SE Scans

A pair of 2D multi-slice SE images with an in-plane isotropic resolution and slice thickness of 1 mm were acquired. In order to cover the entire extent of the brain within a reasonable scan time, 81 slices were acquired using a slice gap of 1 mm. This resulted in a FOV of 256 × 192 × 161 mm^3^. In both cases, TE was 11 ms and FA/refocusing angles were 90°/180°. TR_1_ and TR_2_ were 700 ms and 3000 ms, respectively (Michel et al., 2017). Total scan time was 6 min and 49 s and 9 min and 39 s.

#### DTI 1.5 mm

Diffusion tensor imaging data was acquired using an in-plane isotropic resolution and a slice thickness of 1.5 mm, together with a slice gap of 1 mm. The PA direction was set to be Anterior-Posterior (AP). Fifty six slices were acquired with a multiband acceleration of 2. An in-plane SENSE factor of 2.5 was employed, without Partial Fourier (PF) acceleration. FOV coverage was 256 × 239 × 139 mm^3^. 5 b-values (50, 150, 1000, 1800, and 4500 s/mm^2^) each with 16 directions and one b_0_ image were acquired. FA refocusing angles were 90°/180°, TE = 134 ms and TR = 4899 ms. Note that the water fat shift (WFS) was roughly 20 pixels, which combined to the in-plane spatial resolution of 1.5 mm, made a shift of approximately 3 cm between water and fat. Total scan time was 6 min and 47 s. To correct for susceptibility-induced spatial distortions, two SE echo planar imaging (EPI) images with reversed PA blips and no diffusion weighting were acquired using the same TR/TE combination and readout settings.

#### DTI 1 mm

In one additional case (subject S1), a second DTI dataset was acquired. Parameters were matched to the DTI 1.5 mm protocol in terms of slice gap, b-values/directions, flip/refocusing angles, multiband factor and FOV. Note that the DTI 1 mm protocol was also matched in terms of spatial distortions to the DTI 1.5 mm, since the WFS was 30 pixels and the in-plane resolution 1 mm isotropic. A thinner slice of 1 mm was employed. Other parameters were: number of slices = 70, SENSE factor = 3.5, no PF acceleration, TR/TE = 8808/152 ms, total scan time 12 min and 12 s. Similarly to the DTI 1.5 mm case, two SE EPI images with reverse PA blips but otherwise identical parameters were acquired to correct for susceptibility-induced spatial distortions.

### HF Conductivity Estimation

At HF, the electrical properties of biological tissues depend on their water content (Michel et al., 2017) and ion concentrations (Choi et al., 2020). Magnetic resonance imaging (MRI) allows to derive water content maps (Neeb et al., 2006), enabling tissue characterization in terms of size, type, physiological and/or pathological condition of the cells involved. The water content *W* is related to the T_1_ longitudinal relaxation time according to the following relationship:

(2) $$W=\frac{1}{A+\frac{B}{T_{1}}}$$

where A and B are parameters which depend on the field strength (Fatouros and Marmarou, 1999). Equation 2 states that, in order to derive water content maps, the exact knowledge of the longitudinal relaxation time T_1_ is required. In this study, an alternative approach was employed (Michel et al., 2017), where a transfer function between water maps and a SE T1w image was built. In particular, a SE T1w image is derived from the ratio of two SE images acquired at two repetition times TR_1_ and TR_2_. Such relationship holds:

(3) $$W=w_{1}\,e^{-w_{2}\,I_{r}}$$

where *I_r_* is the image ratio (TR_1_/TR_2_) of the SE image, w=11.525 and w=21.443 are coefficients which were optimized considering typical water concentrations for gray matter (GM), WM, and cerebrospinal fluid (CSF). It is important to note that Equation 2 is optimized for the ratio of SE images acquired at TR_1_ = 700 ms and TR_2_ = 3,000 ms. These were in fact the shortest values that lead to the highest sensitivity of *I_r_* to signal changes in T_1_ for GM, WM, and CSF at 3T (Michel et al., 2017).

From the water content map, by assuming that water concentration univocally pre-determines electrical properties in biological tissues, HF conductivity values can be computed through a second transfer function—which has been optimized considering tissues with large amount of water:

(4) $$\sigma_{H\,F}=c_{1}+c_{2}\,e^{c_{3}\,W}$$

Coefficients were optimized to be c=10.286, c=21.526x10-5, and c=311.852. Note that Equation 4 was defined for water concentrations within the range 0.6 < = *W* < = 1 (Michel et al., 2017) and that receiver gain settings and shimming values were kept the same across scans.

### DTI Pre-processing

The DTI data underwent two levels of pre-processing:

• The first level, which is called “*prep. intermediate*,” includes the denoising procedure described in in Veraart et al. (2016a,b) and the correction for slowly varying receiver bias fields as implemented in MRTricks3 (Tournier et al., 2019).
• The second level, called “*prep. final*,” includes the denoising and bias field procedures just described plus distortion correction and linear registration onto a common anatomical space. To achieve distortion correction, the SE EPI datasets with reversed phase-encode blips are used to estimate the susceptibility induced off-resonance distortion field (Andersson et al., 2003; Smith et al., 2004). These fields are then converted into mm and applied to the DTI data using an interpolation scheme, which considered the effect of the Jacobian. The resulting images are finally rigidly registered (Studholme et al., 1999) onto the space defined by σ_*HF*_ using the IRTK software that was used under License from Ixico Ltd.

### Estimation of LF Variables

To estimate the extracellular volume fraction χ_*e*_, and the intra and extracellular water diffusivities *d_i_* and *d_e_*, the model recently proposed by Lee et al. (2020) was used. This model builds upon the NODDI method, a multi-compartment model widely used to investigate the microstructure of biological tissues (Zhang et al., 2012). NODDI uses three microstructural environments, which are directly related to specific tissue structures, including anisotropic intra and extracellular spaces, and isotropic CSF.

The model works on the DTI signal *S_b_* independent from the choice of the gradient direction applied. In particular, an estimate for *S_b_* (hereafter referred to as ${\bar{S}}_{b}$) is obtained by averaging the *n* acquired gradient directions:

(5) $${\bar{S}}_{b}=\frac{1}{n}\,\sum_{j=1}^{n}S_{b}^{j}$$

Once the average ${\bar{S}}_{b}$ is obtained, the *S_b_* signal can be expressed as a function of NODDI-like sub-variables. These are the intracellular volume fraction *v_ic_*, the isotropic volume fraction *v_iso_*, and the extracellular mean diffusivity *d^∗^_e_*:

$$\frac{S_{b}}{S_{o}}=\left(1-v_{i\,s\,o}\right)\,\left[v_{i\,c}\,e^{-b\,v_{i\,c}\,d_{i\,c}}+\left(1-v_{i\,c}\right)\,e^{-b\,\left(1-v_{i\,c}\right)\,d_{e}^{*}}\right]$$

(6) $$+v_{i\,s\,o}\,e^{-b\,d_{i\,s\,o}}$$

where *S_o_* is the SE EPI signal without diffusion weighting applied, di⁢c=1.7⁢x⁢ 10-3⁢m⁢m2s the intracellular diffusivity, and di⁢s⁢o=3⁢x⁢ 10-3⁢m⁢m2s the isotropic water diffusivity. Note that *d*_*ic*_ and *d*_*iso*_, whose values were gathered from well-established literature (Zhang et al., 2012), were defined *a priori* to stabilize the fit.

The values for *v_ic_*, *v_iso_*, *d^∗^_e_* can be estimated using the following cost function:

(7) $$f=a\,r\,g\,m\,i\,n_{\left(v_{i\,c},v_{i\,s\,o},d_{e}^{*}\right)}\,{||{\bar{S}}_{b}-S_{b}||}_{2}^{2}$$

This is done following the optimized procedure outlined in section “Model Fitting”. The resulting fit is then used to estimate χ_*e*_, *d_e_* and *d_i_*:

(8) $$\chi_{e}=\left(1-v_{i\,s\,o}\right)\,\left(1-v_{i\,c}\right)+v_{i\,s\,o}$$

(9) $$d_{e}=\frac{\left(1-v_{i\,s\,o}\right)\,{\left(1-v_{i\,c}\right)}^{2}\,d_{e}^{*}}{\chi_{e}}+\frac{v_{i\,s\,o}\,d_{i\,s\,o}}{\chi_{e}}$$

(10) $$d_{i}=v_{i\,c}\,d_{i\,c}$$

To compute the extracellular diffusion tensor **D**_*e*_, the following steps were undertaken. The diffusion tensor **D** was estimated at a fixed b-value of 1,000 using the method developed by Veraart et al. (2013) as implemented in MRTricks3 (Tournier et al., 2019). Subsequently, a singular value decomposition (SVD) of the tensor was performed. We denote with*d_xx_*, *d_yy_* and *d_zz_* the computed eigenvalues at each voxel. Finally, under the assumption that **C** and **D**_*e*_share the same eigenvectors (Lee et al., 2020), the following relationship is established:

(11) $${\text{D}}_{e}=\eta \,D$$

where

(12) $$\eta =\frac{3\,d_{e}}{d_{x\,x}+d_{y\,y}+d_{z\,z}}$$

A detailed description on how Equations 8–12 are derived has been recently published (Lee et al., 2020).

### Model Fitting

Minimizing Equation 7 is not straightforward, since in ${\bar{S}}_{b}$ of Equation 5 there are six measurements to fit with only three parameters from Equation 6. Furthermore, the procedure relies on the assumption that ${\bar{S}}_{b}$ is a true representation of *S_b_*. As a result, the fitting procedure was unstable, with the sub-variables *v*_*ic*_, *v*_*iso*_ and *d*^∗^_*e*_ that were consistently underestimated (see section “Estimation of LF Variables” for details).

In this study, the following fitting sub-routine was therefore developed. Instead of fitting *v*_*ic*_, *v*_*iso*_ and *d*^∗^_*e*_ onto ${\bar{S}}_{b}$ of Equation 5 one time, several fitting experiments were performed in parallel. *v*_*ic*_, *v*_*iso*_, and *d*^∗^_*e*_ from Equation 6 were estimated 16 times each time employing a slightly different estimate of ${\bar{S}}_{b}$, which was drawn by choosing *n-1* gradient directions from the *n* available. In this study, *n* = 16, so there were $\left(\begin{array}{c} 16\\ 15 \end{array}\right)$ = 16 unique subsets of gradient directions available. Maximum intensity projection (MIP) images were then calculated by selecting the maximum across the 16 estimates of *v*_*ic*_, *v*_*iso*_, and *d*^∗^_*e*_, independently at each voxel. Initial conditions for each individual run where *v*_*ic*_ = 0, *v*_*iso*_ = 0.5 and *d*^∗^_*e*_ = 0 $\frac{m\,m^{2}}{s}$ (the initial condition of *v*_*iso*_ was empirically set to 0.5, so that the fit converged faster and with fewer iterations). The minimization was carried out by employing a variant of the *fminsearch* function of MATLAB (MathWorks, Natick, MA, United States), which allowed the definition of boundary conditions of the search space. In particular, *v*_*ic*_ and *v*_*iso*_ were constrained to be within the range [0 1], whereas *d*^∗^_*e*_ was forced to be a non-negative quantity. Although the MIP reconstructions improved the overall quality of *v*_*ic*_, *v*_*iso*_ with respect to the individual iterations (see section “Estimation of LF Variables”), some residual artifacts were present in the form of localized darker regions of limited size. After careful inspection of *v*_*ic*_ and *v*_*iso*_, these darker regions were labeled by defining a cut-off value 0.15 below which the maps were assumed to be outside the physiological range (i.e., it is assumed that isotropic water and intracellular volume fractions are always greater than 15% in each voxel). Note that, for *v*_*ic*_, it was also necessary to exclude voxels belonging to the CSF since *v*_*ic*_ must be 0 in these regions. After the labeling was performed, missing values were imputed using a 2D Delaunay triangulation (Delaunay, 1934) in combination with linear interpolation.

### Conductivity Values

To extract the “isotropic equivalent” $\sigma_{L\,F}^{i\,s\,o}$ from the electrical conductivity tensor **C**_*LF*_ (see Figures 1E,F), the following procedure was adopted. An eigenvalue/eigenvector decomposition of **C**_*LF*_ at each voxel was carried out. We denote the eigenvalues at a particular voxel location as *c*_*xx*_, *c*_*yy*_ and *c*_*zz*_. The “isotropic equivalent” conductivity *c*_*iso*_ is computed as $c_{i\,s\,o}={\left(c_{x\,x}\,c_{y\,y}\,c_{z\,z}\right)}^{\frac{1}{3}}$. Thus, *c*_*iso*_ represents the conductivity value required to generate an isotropic tensor whose volume matches that of the measured ellipsoids. Repeating this procedure at every location lead to $\sigma {}_{L\,F}^{i\,s\,o}$.

Following the calculation of $\sigma_{L\,F}^{i\,s\,o}$, the segmentation of the brain structures was performed according to the following procedure. At first, brain extraction of the MP-RAGE data was performed using the brain extraction tool (BET) of FSL (Smith, 2002). The brain extracted data was subsequently rigidly registered and re-sampled onto the space defined by $\sigma_{L\,F}^{i\,s\,o}$ using IRTK and segmented into WM, GM and CSF tissue classes using the FMRIB’s Automated Segmentation Tool (FAST) of FSL (Zhang et al., 2001). In order to reduce partial volume effects, a threshold value of 99% was applied to the probability maps returned by FAST. The obtained eroded masks were then used to segment WM and GM. In order to segment the CSF, a threshold value of $2\,\frac{S}{\text{m}}$ was applied directly on σ_*HF*_, since the conductivity values are considerably higher than those of the GM and WM (see Figure 1B). We indeed observed better performances than the MP-RAGE segmentation, especially in the apical regions of brain, where the interfaces between CSF and GM are thin.

Gray matter, WM, and CSF masks were employed to segment $\sigma_{L\,F}^{i\,s\,o}$. We denote with *ϕ_k_* the LF isotropic conductivity distribution of tissue type *k*, with *k* ∈ (*GM, WM, CSF*). To assess distribution properties, we compute its mean (*mean_ϕk_*), standard deviation (*std_ϕk_*), and coefficient of variation (*CV_ϕk_* = $\frac{s\,t\,d_{\phi_{k}}}{m\,e\,a\,n_{\phi_{k}}})$. To ascertain the amount of overlap between GM and WM distributions, we employ the coefficient of joint variation $CJV_{\left(\phi GM,\phi WM\right)}=\frac{std_{\phi GM}+std_{\phi WM}}{mean_{\phi GM}-mean_{\phi WM}}$ (Ganzetti et al., 2016). Note that this was not performed for combinations of tissues containing the CSF, since its conductivity values are considerably higher than GM and WM and there is no overlap.

### Resolution Test

To measure the achieved spatial resolution of the CTI maps, the following two steps were carried out.

First, we assessed in one subject, i.e., S1, the differences in $\sigma_{L\,F}^{i\,s\,o}$ obtained with the DTI 1 mm vs. DTI 1.5 mm protocols (hereafter referred to as σL⁢F,D⁢T⁢I⁢ 1⁢m⁢mi⁢s⁢o and σL⁢F,D⁢T⁢I⁢ 1.5⁢m⁢mi⁢s⁢o, see section “Image Acquisition” for details). In particular, *ϕ*_*k*_ was computed for GM, WM and CSF tissue classes. To assess the capability of the two maps to distinguish GM and WM, *CJV_(ϕGM,ϕWM)_* was calculated.

To investigate whether σL⁢F,D⁢T⁢I⁢ 1⁢m⁢mi⁢s⁢o had higher spatial resolution than σL⁢F,D⁢T⁢I⁢ 1.5⁢m⁢mi⁢s⁢o, the power spectral density (PSD) along the PA direction was computed. The resulting spectra were then averaged and converted into a decibel (dB) scale. Please note that in order to normalize the spectra, the DC component in the dB scale was removed in all cases.

A second test aimed at assessing the spectral spatial properties of σL⁢F,D⁢T⁢I⁢ 1.5⁢m⁢mi⁢s⁢o relative to the conductivity at HF, i.e., σ_*HF*_. To do so, a second PSD was computed on σ_*HF*_ (hereafter referred to as σ_*HF*, 1*mm*_). To further substantiate the potential loss in spatial resolution of σL⁢F,D⁢T⁢I⁢ 1.5⁢m⁢mi⁢s⁢o with respect to σ_*HF*, 1*mm*_, two new reference images at a lower resolution were obtained by: (i) down sampling the original σ_*HF*, 1*mm*_ to 1.5 mm and 2 mm in-plane resolution, respectively, and (ii) by up sampling them again to match the original grid-size of 1 mm. On these lower resolution images (σ_*HF*, 1.5*mm*_ and σ_*HF*, 2*mm*_, respectively), new PSDs were computed.

## Results

### DTI Pre-processing

**FIGURE 2:**
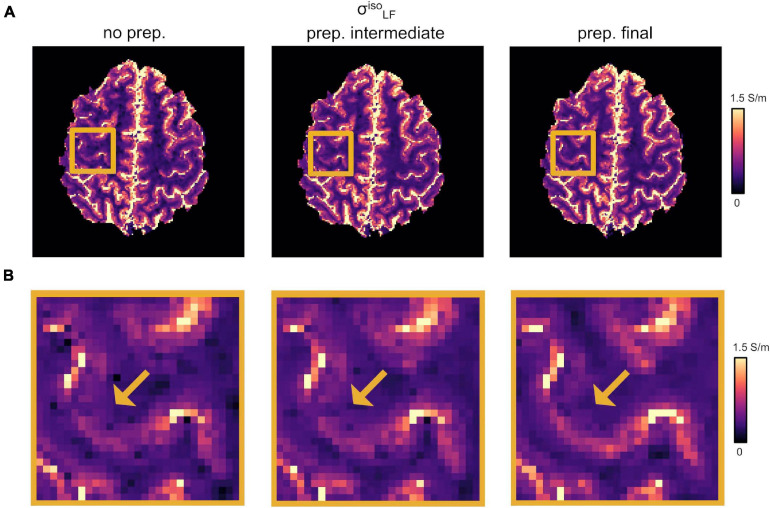
Effect of DTI pre-processing. **(A)** Changes in $\sigma_{L\,F}^{i\,s\,o}$ as a function of the different DTI pre-processing steps. (Left) No pre-processing applied (“*no prep.”*). (Center) Intermediate pre-processing applied (“*prep. intermediate*”); i.e., denoising plus bias field correction). (Right) Full pre-processing applied (“*prep. final*”); i.e., “*prep. intermediate*” plus distortion correction and linear registration onto σ_*HF*_. **(B)** Magnified region from panel **(A)**. Yellow arrows point to WM/GM boundaries.

Our optimized DTI pipeline lead to an improved definition of the conductivity maps, especially at the brain tissue interfaces in the apical regions. Figure 2A reports the changes in $\sigma_{L\,F}^{i\,s\,o}$ as a function of the different DTI pre-processing steps described in section “DTI Pre-processing.” Figure 2 shows incremental improvements following the application of the denoising step together with the correction for slowly varying receiver bias fields, i.e., “*prep. intermediate*,” and distortion correction plus linear registration to the common anatomical space, i.e., “*prep. final*.” A more accurate co-registration to the anatomical image improves the overlap between structural properties and HF conductivity values. Accordingly, the LF conductivity pattern of the GM emerged from the darker WM of the background (Figure 2B, yellow arrows).

### Estimation of LF Variables

**FIGURE 3:**
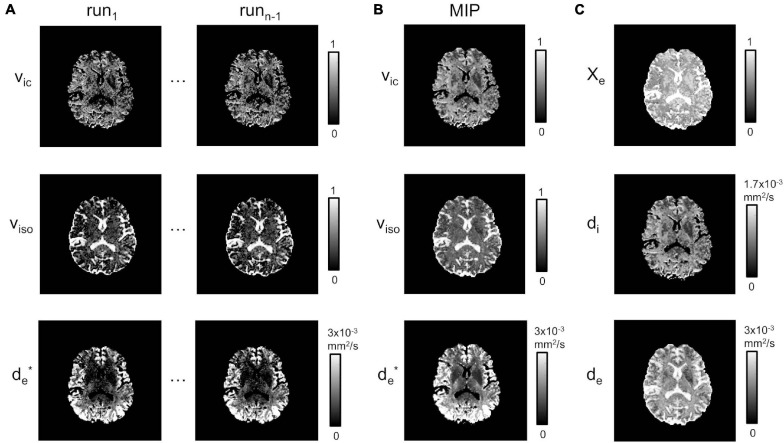
Model estimation. **(A)** Estimated *v_ic_*, *v_iso_* and $d_{e}^{*}$ at runs 1 to *n-1*. **(B)** MIP projections for *v*_*ic*_, *v*_*iso*_ and $d_{e}^{*}$ (plus Delaunay correction). **(C)** χ_*e*_, *d_i_* and *d_e_*.

Figure 3A shows the estimates for *v*_*ic*_, *v*_*iso*_ and *d*^∗^_*e*_ chosen for two runs of the fitting procedure described in section “Model Fitting”. While these appear reproducible and stable over the CSF, the fitting is not stable in the WM and in the GM regions, where isolated and/or clusters of pixels for both *v*_*ic*_ and *v*_*iso*_ maps are underestimated or close to zero. For *d*^∗^_*e*_, instead, estimates appear to be acceptable in the cortical areas, but they are subject to noise enhancement deeper in the brain, where the anatomy is farther away from the receiver coils and therefore the SNR lower. MIP maps (plus Delaunay correction) for *v*_*ic*_, *v*_*iso*_ and *d*^∗^_*e*_ are displayed in Figure 3B.

χ_*e*_, *d_e_*, and *d_i_* estimates from Equations 8–10 are shown in Figure 3C. As expected, χ_*e*_ showed values close to one in areas corresponding to the CSF, while *d_i_* and *d_e_* had richer information, depending on the presence of higher fiber directionality and/or water content.

Finally, an example of the estimated extracellular diffusion tensor **D**_*e*_calculated using Equations 11, 12 is provided in Figure 1D.

### Resolution Test and Conductivity Values

**FIGURE 4:**
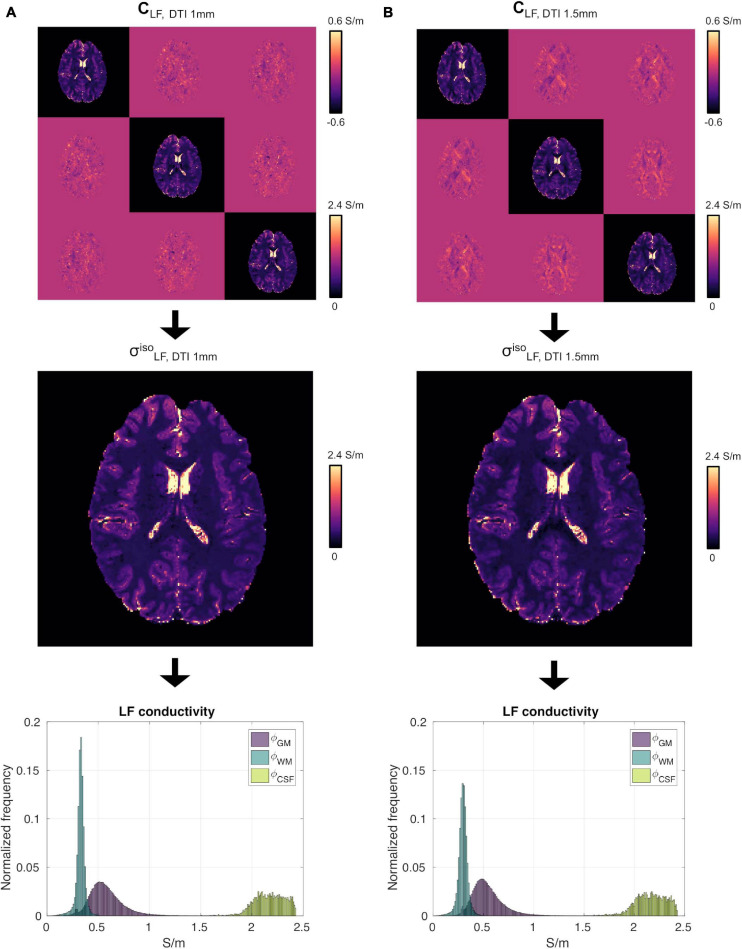
High-resolution experiment on subject S1. **(A)** **C**_*LF*_ (top), $\sigma_{L\,F}^{i\,s\,o}$ (center) and *ϕ_GM_*, *ϕ_WM_* and *ϕ_CSF_* (bottom) reconstructed using the DTI 1 mm protocol. Panel **(B)** same as **(A)** using the DTI 1.5 mm protocol data.

We did not observe strong qualitative differences in the conductivity tensor obtained using the DTI 1 mm and DTI 1.5 mm protocols (**C**_*LF, DTI 1 mm*_ vs. **C**_*LF, DTI 1.5 mm*_, Figure 4, top) nor in $\sigma_{LF,DTI1mm}^{iso}$ vs $\sigma_{LF,DTI1.5mm}^{iso}$ (Figure 4, center). Tissue distributions were also similar (Figure 4, bottom). From a quantitative standpoint, mean and standard deviations of *ϕ_GM_*, *ϕ_WM_* and *ϕ_CSF_* for S1 were 0.58 ± 0.18 $\frac{S}{m}$, 0.33 ± 0.05 $\frac{S}{m}$, and 2.17 ± 0.15 $\frac{S}{m}$ for σL⁢F,D⁢T⁢I⁢ 1⁢m⁢mi⁢s⁢o, and 0.55 ± 0.17 $\frac{S}{m}$ 0.30 ± 0.05 $\frac{S}{m}$, and 2.16 ± 0.15 $\frac{S}{m}$ for σL⁢F,D⁢T⁢I⁢ 1.5⁢m⁢mi⁢s⁢o. Finally, σL⁢F,D⁢T⁢I⁢ 1.5⁢m⁢mi⁢s⁢o had a similar capacity to distinguish GM from WM as compared to σL⁢F,D⁢T⁢I⁢ 1⁢m⁢mi⁢s⁢o (*CJV*_(*ϕGM*_,*ϕ*_*WM*_) = 91.48/92.57).

Figure 6A reports the reconstructions for σ_*HF*, 1*mm*_, σ_*HF*, 1.5*mm*_, σ_*HF*, 2*mm*_ and σL⁢F,D⁢T⁢I⁢ 1.5⁢m⁢mi⁢s⁢o in subject S5. The PSDs for all other subjects are shown in Figure 6B.

**FIGURE 5:**
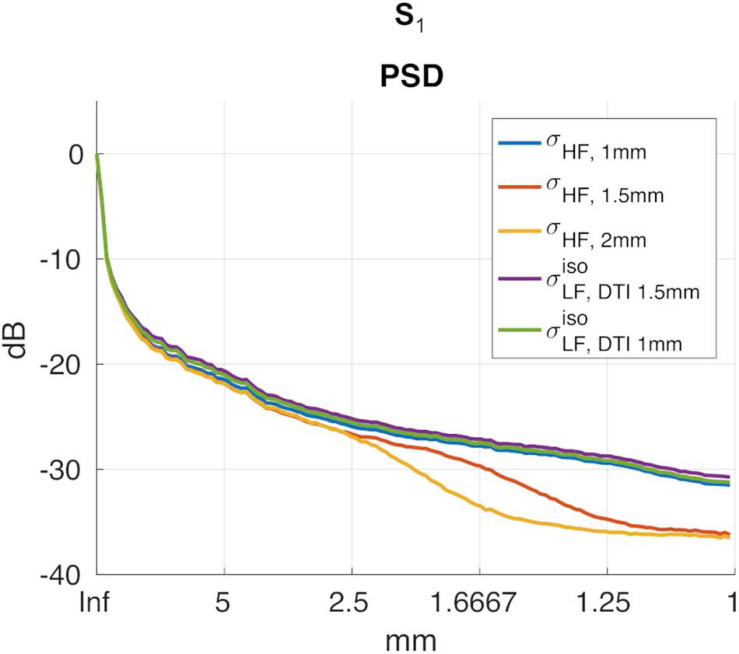
PSDs for σ_*HF*, 1*mm*_, σ_*HF*, 1.5*mm*_, σ_*HF*, 2*mm*_, σL⁢F,D⁢T⁢I⁢ 1.5⁢m⁢mi⁢s⁢o and σL⁢F,D⁢T⁢I⁢ 1⁢m⁢mi⁢s⁢o in subject S1.

**FIGURE 6:**
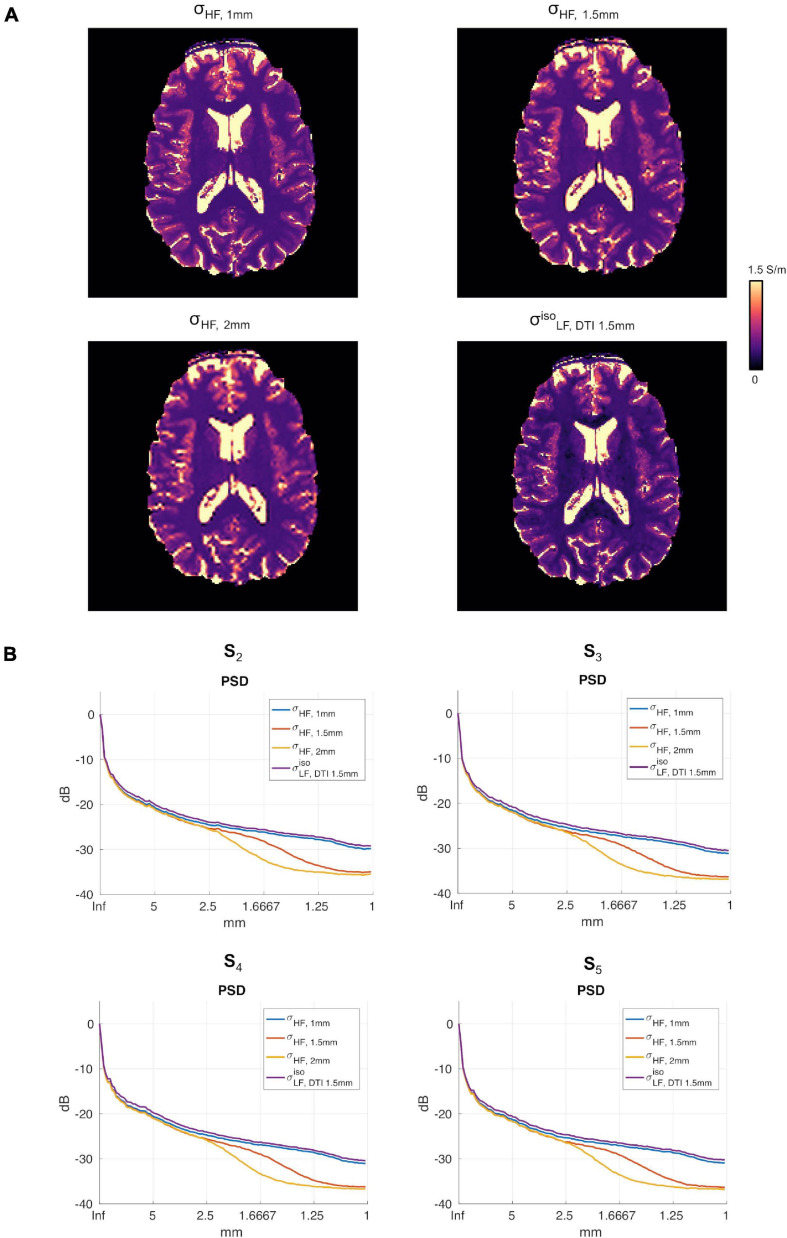
**(A)** Conductivity maps in subject S5 for σ_*HF*, 1*mm*_, σ_*HF*, 1.5*mm*_, σ_*HF*, 2*mm*_ and σL⁢F,D⁢T⁢I⁢ 1.5⁢m⁢mi⁢s⁢o in the apical regions of the brain. **(B)** σ_*HF*, 1*mm*_, σ_*HF*, 1*mm*_, σ_*HF*, 2*mm*_, σL⁢F,D⁢T⁢I⁢ 1.5⁢m⁢mi⁢s⁢o PSDs for subjects S2 to S5.

**FIGURE 7:**
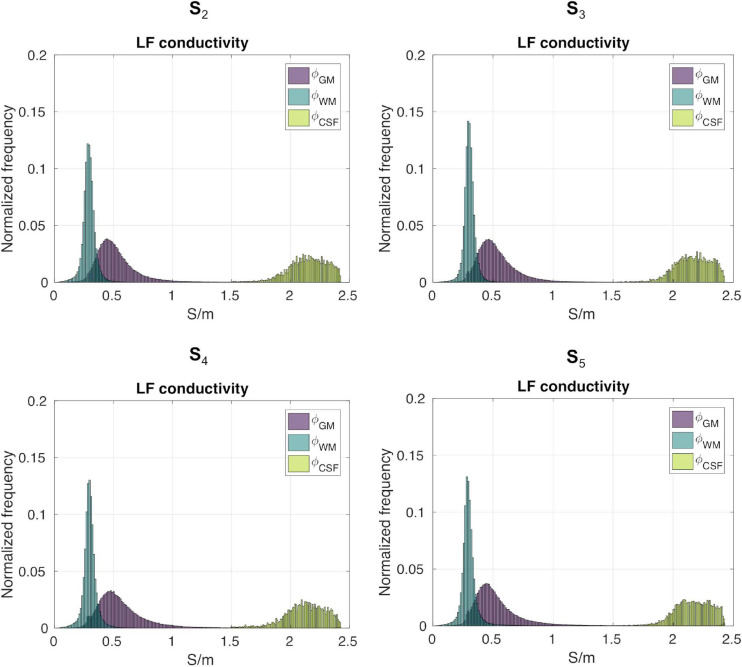
*ϕ_GM_*, *ϕ_WM_* and *ϕ_CSF_* distributions for subjects S2 to S5.

Figure 7 shows the distributions of *ϕ_k_* obtained in subjects S2 to S5. Table 1 reports *mean_ϕk_* and *std_ϕk_* in GM, WM and CSF, at LF and HF. We obtained similar values across subjects, with conductivity values for WM, GM and CSF of 0.55 ±0.01$\frac{S}{\text{m}}$, 0.3 ± 0.01$\frac{S}{\text{m}}$ and 2.15 ± 0.02$\frac{S}{\text{m}}$ at LF, and of 0.63 ±0.02$\frac{S}{\text{m}}$, 0.37 ± 0.01$\frac{S}{\text{m}}$ and 2.19 ± 0.01$\frac{S}{\text{m}}$ at HF. Table 2 reports *mean* and *std* for the diagonal components of the conductivity tensor in GM, WM and CSF at LF. Finally, Table 3 shows *CV_ϕk_* in GM, WM and CSF and *CJV_(ϕGM,ϕWM)_* at LF/HF in all subjects that were imaged.

**TABLE 1:** *Mean_ϕk_* and *std_ϕk_* in GM, WM and CSF, at LF (left) and HF (right) in all subjects.

	**Me*an*±s*t*d_*ϕ*G*M*_**		**Me*an*±s*t*d_*ϕ*W*M*_**		**Me*an*±s*t*d_*ϕ*C*SF*_**	
	**$\sigma_{\mathrm{LF}}^{\mathrm{iso}}$**	**σ_*HF*_**	**$\sigma_{\mathrm{LF}}^{\mathrm{iso}}$**	**σ_*HF*_**	**$\sigma_{\mathrm{LF}}^{\mathrm{iso}}$**	**σ_*HF*_**
S_1_	0.55 ± 0.17 S/m	0.63 ± 0.19 S/m	0.30 ± 0.05 S/m	0.36 ± 0.05 S/m	2.16 ± 0.15 S/m	2.19 ± 0.15 S/m
S_2_	0.53 ± 0.20 S/m	0.61 ± 0.22 S/m	0.29 ± 0.06 S/m	0.36 ± 0.05 S/m	2.15 ± 0.16 S/m	2.19 ± 0.16 S/m
S_3_	0.54 ± 0.19 S/m	0.62 ± 0.21 S/m	0.30 ± 0.05 S/m	0.36 ± 0.04 S/m	2.16 ± 0.15 S/m	2.20 ± 0.15 S/m
S_4_	0.57 ± 0.24 S/m	0.66 ± 0.27 S/m	0.30 ± 0.08 S/m	0.37 ± 0.08 S/m	2.12 ± 0.18 S/m	2.18 ± 0.17 S/m
S_5_	0.55 ± 0.27 S/m	0.64 ± 0.29 S/m	0.31 ± 0.17 S/m	0.39 ± 0.17 S/m	2.17 ± 0.15 S/m	2.20 ± 0.15 S/m

**TABLE 2:** *Mean* and *std* of the diagonal components of the conductivity tensor in GM, WM, and CSF at LF in all subjects.

		***c_xx_***	***c_yy_***	***c_zz_***
*GM*	S_1_	0.57 ± 0.19 S/m	0.56 ± 0.18 S/m	0.53 ± 0.18 S/m
	S_2_	0.56 ± 0.22 S/m	0.55 ± 0.22 S/m	0.53 ± 0.22 S/m
	S_3_	0.56 ± 0.20 S/m	0.56 ± 0.20 S/m	0.53 ± 0.21 S/m
	S_4_	0.60 ± 0.26 S/m	0.60 ± 0.26 S/m	0.56 ± 0.26 S/m
	S_5_	0.57 ± 0.27 S/m	0.57 ± 0.28 S/m	0.54 ± 0.28 S/m
*WM*	S_1_	0.32 ± 0.09 S/m	0.34 ± 0.10 S/m	0.33 ± 0.10 S/m
	S_2_	0.32 ± 0.10 S/m	0.32 ± 0.10 S/m	0.32 ± 0.11 S/m
	S_3_	0.31 ± 0.09 S/m	0.34 ± 0.09 S/m	0.33 ± 0.09 S/m
	S_4_	0.32 ± 0.11 S/m	0.34 ± 0.11 S/m	0.33 ± 0.12 S/m
	S_5_	0.33 ± 0.19 S/m	0.36 ± 0.19 S/m	0.34 ± 0.19 S/m
*CSF*	S_1_	2.20 ± 0.14 S/m	2.17 ± 0.19 S/m	2.14 ± 0.19 S/m
	S_2_	2.21 ± 0.15 S/m	2.16 ± 0.21 S/m	2.13 ± 0.22 S/m
	S_3_	2.20 ± 0.14 S/m	2.17 ± 0.19 S/m	2.14 ± 0.19 S/m
	S_4_	2.21 ± 0.15 S/m	2.15 ± 0.22 S/m	2.09 ± 0.21 S/m
	S_5_	2.21 ± 0.14 S/m	2.19 ± 0.19 S/m	2.13 ± 0.19 S/m

**TABLE 3:** *CV_ϕGM_*, *CV_ϕWM_*, *CV_ϕCSF_* and *CJV_(ϕGM,ϕWM)_* in all subjects at LF (left) and HF (right).

	***CV_ϕGM_***		***CV_ϕWM_***		***CV_ϕCSF_***		***CJV_(ϕGM,ϕWM)_***	
	**$\sigma_{\mathrm{LF}}^{\mathrm{iso}}$(%)**	**σ_*HF*_(%)**	**$\sigma_{\mathrm{LF}}^{\mathrm{iso}}$(%)**	**σ_*HF*_(%)**	**$\sigma_{\mathrm{LF}}^{\mathrm{iso}}$(%)**	**σ_*HF*_(%)**	**$\sigma_{\mathrm{LF}}^{\mathrm{iso}}$(%)**	**σ_*HF*_(%)**
S_1_	31.52	30.40	18.41	12.61	7.08	6.79	91.48	89.83
S_2_	37.62	35.34	20.35	13.53	7.65	7.32	109.61	105.54
S_3_	35.94	34.66	16.52	11.39	6.92	6.67	104.20	100.06
S_4_	42.48	41.09	25.45	20.93	8.23	7.66	117.30	119.05
S_5_	48.84	45.42	54.69	45.32	6.88	6.64	188.78	183.59

## Discussion

In this study, we propose a framework to perform CTI of the human brain without relying on the Laplacian operator of MR-EPT methods. This was achieved by combining HF conductivity mapping based on water maps with multi b-value DTI data.

Traditional CTI methods estimate the conductivity values at HF by means of phase-based MR-EPT techniques (Sajib et al., 2018; Jahng et al., 2020; Lee et al., 2020). Whilst reliable and reproducible conductivity values can be obtained using phase-based MR-EPT (Mandija et al., 2020), there are limitations connected to the use of the Laplace operator, which is noise sensitive and less reliable at tissue boundaries. Several studies aimed at improving image quality of MR-EPT maps and to compensate for boundary artifacts by making assumptions on the trans-receiving phase of the MR signal (Van Lier et al., 2012; Seo and Woo, 2014), and by implementing optimized smoothing operators (Ropella and Noll, 2017; Sajib et al., 2018; Jahng et al., 2020). Though all this work successfully tackled most of these challenges, MR-EPT methods are generally characterized by modest to low spatial resolution levels. In fact, when considering the nominal resolution without accounting for the effect of the Laplace operator and/or other smoothing kernels, which extend blurring (Van Lier et al., 2012), typically reported values have been 1.6 × 1.6 × 2 mm^3^ (Voigt et al., 2011), 1.7 × 1.7 × 1.7 mm^3^ (Gurler and Ider, 2017) and 1.87 × 1.87 × 4 mm^3^(Katoch et al., 2018).

For these reasons, we propose an alternative strategy for the estimation of the conductivity at HF (Michel et al., 2017). In particular, water maps are estimated using a set of deterministic equations applied to SE data, which are then used to calculate a conductivity map. Because this is done on a pixel-by-pixel basis (as opposed to MR-EPT where the Laplace operator is typically defined as a convolution kernel in 3D), the achieved spatial resolution of the conductivity HF maps and of the SE data should be very close. Moreover, since the SE sequences are acquired in steady state, there is no spatial blurring introduced by signal decay as it is for non-steady state sequences (Brown et al., 2014). Thus, by employing the proposed method, it is possible to attain an actual in-plane isotropic resolution for σ_*HF*_ of 1 mm with a slice thickness of 1 mm (note that, in this context, the achieved resolution should be independent from the choice of the slice gap, since σ_*HF*_ is retrieved solely from the ratio of two SE images acquired in 2D on a pixel-by-pixel basis).

By designing a framework independent from the Laplace operator, our approach could effectively discriminate boundaries between different brain tissues, both in terms of conductivity values and fiber orientations, and to pave the way for the use of CTI maps to several applications. These include (although they are not restricted to) electroencephalography/magnetoencephalography (EEG/MEG) source imaging (Cho et al., 2015; Liu et al., 2017; Marino et al., 2019). To perform accurate EEG/MEG source imaging, a realistic head model of the subject undergoing the experimental investigation is needed (Cho et al., 2015; Morales et al., 2019). This head model is derived from a whole-head image of the subject, and it relies on multiple sources of information including electrode positions (Marino et al., 2016; Taberna et al., 2019), tissue geometry (Taberna et al., 2021), and conductive properties. With this work, we aim at contributing to the optimization of the latest aspect, by proposing a methodology for subject-specific head-modeling. In particular, as the reference head geometry is typically provided by a high-resolution anatomical scan, the possibility of achieving a comparable resolution with CTI maps might positively impact the accuracy of source imaging reconstructions. In fact, whilst previous literature showed that anisotropy can be built into a head model (Morales et al., 2019), an approach to extract local conductivity information on a voxel by voxel basis is still an open issue. In fact, conventional head models are built using fixed conductivity values, often taken from the literature (Michel et al., 2004) following tissue segmentation into well-defined tissue compartments. By including anisotropy and voxel-wise conductivity information, the accuracy of the EEG/MEG head modeling could increase, hence providing more accurate source estimates not only in healthy subjects but also for those individuals with altered structural and electrical brain properties such as neurological patients.

In CTI imaging, the DTI data plays a crucial role in the determination of the conductivity tensor. Ideally, the spatial resolution of σ_*HF*_ and of the DTI data should coincide. However, it is challenging to acquire DTI data at 1 mm in-plane isotropic resolution due to the prolonged scan times and increased spatial distortions. In this study, the SENSE factor of the high-resolution protocol was increased to match the two acquisitions in terms of spatial distortions, although this amplified thermal/g-factor noise (Pruessmann et al., 1999). Moreover, high-resolution DTI protocols often lead to prolonged TEs. This is particularly relevant for the acquisitions of CTI maps, where a short TE is preferable but not achievable since DTI scans with very large b-values are needed to separate extra and intra-cellular diffusion components (Clark et al., 2002). All these aspects, combined to the higher resolution of DTI 1 mm protocol, meant that the second acquisition had a low SNR.

To assess the impact that the resolution of the DTI data had on the proposed reconstruction framework, two DTI datasets at different resolutions were acquired. We found small differences between the conductivity maps at LF obtained using DTI data at 1.5 vs. 1 mm in-plane isotropic resolution. This was assessed in a dual manner. First, we compared the final reconstructions, both in terms of the capacity of distinguishing between brain tissues, with a special focus on GM and WM, and of the conductivity values, which were found to be similar between methods and in line with previous work (Jahng et al., 2020). Second, we compared the PSD in the calculated $\sigma_{L\,F}^{i\,s\,o}$ maps, which showed similar spectral content properties (Figure 5). We also investigated the impact of conductivity maps at HF resampled at a lower resolution than 1 mm, and noticed that the spectral content indeed decreases (see Figures 5, 6B). These aspects, which were marginally explored in previous literature, makes of utmost importance the choice of the approach to achieve HF information, which directly affects the maximum achievable spatial resolution. In particular, these findings suggest that while the spatial resolution of the CTI maps is mainly driven by the resolution of the HF conductivity map, the fit from the DTI data acts as a conductive dumping operator, which mediates the relationship between HF and LF, but without introducing substantial spatial smoothing. Accordingly, the proposed framework, which links its resolution to the ones of the SE images, seems to be a powerful alternative to conventional CTI methods that make use of phase-based MR-EPT.

While previous CTI (Tuch et al., 2001; Sajib et al., 2018; Lee et al., 2020) studies pioneered the design of the framework currently employed to map conductivity *in vivo* at low frequencies, the pre-processing of the DTI data, which included at most a rigid registration step (Jahng et al., 2020), has not been considered. In this work, we showed that an accurate pre-processing of the DTI data is important to augment the quality of the conductivity tensor. In particular, a DTI pipeline should ameliorate the estimated diffusion coefficients, since the combination of multi-exponential curves in the water diffusivity model is severely affected by noise (Tuch et al., 2001; Sajib et al., 2018; Jahng et al., 2020; Lee et al., 2020), and improve the spatial correspondence between SE and DTI data, since the latter is distorted. Thus, special attention was paid toward the definition of a pre-processing procedure, which included denoising, bias field correction, distortion correction and rigid registration to the space defined by σ_*HF*_. The application of the proposed pipeline resulted in consistent improvements, especially at the brain tissue interfaces of $\sigma_{L\,F}^{i\,s\,o}$ between GM and WM and between GM and CSF (Figure 2).

This was combined with an optimized routine to improve the fitting of the multi-compartment NODDI model, which consisted of multiple runs of the fitting, depending on the number of directions used in the DTI data, oriented toward the definition of MIP images for χ_*e*_, *d_i_* and *d_e_*. These MIP images resulted in stable estimates for the intracellular and isotropic volumes, and the extracellular diffusivity, which all intervene in the definition of LF variables integrated by Equation 1. In contrast, the same quantities reconstructed using the same model were generally artifacted and/or underestimated in previous studies (Lee et al., 2020), especially *d_e_* over GM and WM regions. In this study, we found a similar behavior when looking at the estimation for each run (Figure 3A). However, these artifacts were largely removed when considering the MIP reconstructions together with the Delaunay interpolation (Figure 3B). In particular, *v*_*ic*_ was close to zero in the CSF areas. Conversely, it was greater in GM and WM where there is the co-existence of cells and extracellular matrix. *v*_*iso*_ was close to one in the CSF, where no fibers are present, and had a lower value in GM and WM. For *d*^∗^_*e*_, the diffusivity was higher in GM and lower in WM, which is in line with a more highly fiber-packed environment characterizing WM as compared to GM. Furthermore, it was close to zero over the CSF since the diffusivity is predominantly isotropic.

When looking at the achieved conductivity values, our findings highlighted reproducible measurements within- and between scans for each of the considered tissues, which is expected in healthy subjects, but less in patients. Note that the weighting introduced by the DTI is stronger in the GM and WM regions compared to the CSF, because of their complicated tissue structures (Le Bihan, 2003). Namely, lower conductivity values are observed for $\sigma_{L\,F}^{i\,s\,o}$ compared to σ_*HF*_ because of the highly anisotropic environment. Instead, the conductivity values in the isotropic CSF did not change significantly between LF and HF (Lee et al., 2020).

Maps are expected to be sensitive to a variety of pathological conditions, such as stroke and tumors (Katscher et al., 2013; Shin et al., 2015; Balidemaj et al., 2016a, b; Jensen-Kondering et al., 2020). Moreover, they could also be employed to study other conditions such as neurodegenerative diseases etc.

This study has several limitations. Concerning the HF conductivity maps, we did not correct for the inhomogeneities of the transmit B1^+^ field, which affects the flip angle and so the acquired signal, especially in the center of the FOV of brain scans. This might have introduced errors in the calculation of the water maps, especially in the ventricles (Michel et al., 2017). In addition, this source of bias is made stronger by the inherent relationship between water content and conductivity values reported in Equation 4, which is non-linear with derivative $\frac{d\,\sigma_{H\,F}}{d\,W}\propto {\text{e}}^{{\text{c}}_{3}\,\text{W}}$ (Michel et al., 2017). Thus, water estimation uncertainties in brain regions with high water content such as the CSF will be subject to a larger propagation of errors, explaining why in Figures 5, 7 there was a large spreading of the CSF conductivity values. In this study, we did not explicitly account for B1^+^ inhomogeneities. However, it is clear that for an accurate estimation of the CSF conductivity values, residual sources of bias such as the B1 transmit field should be taken into account.

*I_r_* from Equation 3 is calculated as a ratio of two separate SE images. This might be sensitive to motion and other sources of inconsistency between scans. Thus, a more rigorous approach would include the measurement of a fast T_1_ map (Deoni et al., 2005) as opposed to taking image ratios. The former could in fact be employed to retrieve a water content map by means of Equation 2.

In this study, the scan time of the SE and for the DTI acquisitions was long. Therefore, a slice gap of 1 mm was employed for their acceleration. As mentioned previously, σ_*HF*_ is retrieved from the ratio of 2D SE images performed on a slice-by-slice basis and so the spatial resolution is preserved. However, the introduction of a non-negative slice gap compromises head coverage, which was, at least for the case of the SE data, halved. Thus, future studies should be oriented toward the re-optimization of the procedure outlined in Michel et al. (2017) using a faster acquisition scheme in full 3D. Other approaches for the acceleration of the DTI acquisition should be employed as well to ameliorate brain coverage whilst improving scanning efficiency.

NODDI has been widely employed to estimate neurite density. However, it is characterized by some simplifying assumptions, including the description of the orientation of the axons which does not consider fiber crossing (Schmahmann et al., 2009). Furthermore, NODDI employs fixed values for the intracellular and isotropic diffusivities (*d*_*ic*_ and *d*_*iso*_ of Equation 6). Whilst these values are established in the literature, other choices are possible. For example, the multi-compartment spherical mean technique (SMT) model (Kaden et al., 2016a,b), which was recently employed in CTI imaging (Jahng et al., 2020), assesses intrinsic diffusivity on a voxel by voxel basis. In particular, this model describes the microstructural environment using two compartments (as opposed to three compartments as it is for NODDI), i.e., the intra- and extra-neurite environments (Kaden et al., 2016a,b). By taking into account these aspects, a voxel-wise estimate of the intrinsic diffusivities is possible, which could allow for a potentially more accurate implementation of the CTI framework. Future experimental work should be oriented toward the development and validation of techniques for their estimation (Jahng et al., 2020). This, which may be an important aspect to consider in healthy subjects, could become a critical factor when extending the application of the proposed method into the clinical environment.

The value of the ratio of ion concentrations between intracellular and extracellular spaces (parameter β, Equation 1) was assumed to be 0.41 (Sajib et al., 2018). Whilst methods to experimentally determine β using MRI are still not available, this value has been measured accurately using other technology, and specifically by looking at intra- and extracellular Na^+^, Cl^–^, K^+^, and Ca^2 +^ ion-concentration levels with microelectrodes (Hansen, 1985; Volkov et al., 1997; Katoch et al., 2018; Sajib et al., 2018).

Our approach based on water-mapping techniques assumes that the electrical conductivity at HF is pre-determined by tissue water-content (Michel et al., 2017). This can be valid up to a certain extent, when extending the application of our approach to clinical application, this assumption might not hold, especially for those pathologies characterized by unbalanced ion concentrations such as neurodegenerative disease (Reetz et al., 2012; Petracca et al., 2016; Huhn et al., 2019). In fact, it is known that electrical conductivity changes with ion concentrations and mobility (Gabriel et al., 1996a; Grimnes and Martinsen, 2000; Choi et al., 2020), and the relationship between water concentration and ion mobility is not straightforward. Thus, thorough experimental validation using conductivity phantoms should be performed to test the validity of such assumption (Choi et al., 2020).

However, there is some initial experimental evidence which suggests that higher water content within the brain is also associated with higher conductivity values. In this work (Kurtzbard et al., 2021), the authors measured electrical conductivity and permittivity in 50 neonates scanned as part of The Developing Human Connectome Project (Makropoulos et al., 2018). This study is relevant since: (i) the infant brain presents higher water concentration levels than the adult brain (note that, in this context, the higher water content is also responsible for the longer NMR relaxation properties of the infant brain (Ferrazzi et al., 2018), (ii) the authors tested their technique on a 3T system, and (iii) they employed MR-EPT (Marques et al., 2015). Although the reported variability across subjects was relatively high, this study showed how the neonatal brain conductivity values were, on average, 1.8 times greater than in the adult brain. MR-EPT is thought to be predominantly sensitive to changes in ions concentration and mobility (Choi et al., 2020). However, these initial results suggest that there might be an explicit dependency between water concentration and conductivity values, thus partly justifying the assumptions which were made in this study to overcome the intrinsic limitations of MR-EPT methods.

The primary objective of this study was to provide a framework to perform CTI mapping *in vivo* without MR-EPT in a robust way. By using the proposed technique, we were able to achieve reproducible results over a population of five healthy subjects scanned at rest. However, accuracy of the conductivity measurement is also important. To validate our technique, we relied on well-established literature, mostly in relation to the estimation of σ_*HF*_ (Michel et al., 2017), which was in turn based on a large body of work (Gabriel et al., 1996b; Whittall et al., 1997; Neeb et al., 2006; Abbas et al., 2015), reporting strong value correspondences with the target tissues. However, there is an inherent large variability in the field of conductivity mapping; for example, CSF conductivity values were found to be within the range 1 to 2.51 $\frac{S}{\text{m}}$ in a recent systematic review (McCann et al., 2019). Thus, it is clear that further validation using phantoms with known conductivity values and/or numerical simulations are warranted to validate the proposed technique. Note that, in this context, the proposed framework is flexible, as Equation 4—which links brain water content to conductivity values—could be re-optimized each time definite reference conductivity values are available. Still, there was close agreement between our results and CTI literature validated on phantoms and *in vivo* (Jahng et al., 2020), citing 0.52 $\frac{S}{\text{m}}$ and 0.27 $\frac{S}{\text{m}}$ for GM and WM tissues. Our results, which directly derive from established reference conductivity values (Michel et al., 2017), are compatible with reference *ex vivo* studies for which measurements of 0.59, 0.34, and 2.14 $\frac{S}{\text{m}}$ were reported in GM, WM, and CSF (Gabriel et al., 1996b). They are also in line with a large body of literature concerning non-invasive conductivity mapping (McCann et al., 2019). Nonetheless, it is stressed that the conductivity values achieved in this study *should not directly be used as reference*, as a thorough validation [see for example the work performed in Choi et al. (2020)] has not been performed. In this context, future work with dedicated physical phantoms should be oriented toward employing different reference values for each target tissue. Thus, while delivering a framework that provides reproducible and high-quality CTI maps to overcome several limitations of MR-EPT has been achieved, we assert that a validation is warranted in order to test the reliability of the proposed technique and to benchmark the obtained results.

## Data Availability Statement

The raw data supporting the conclusions of this article will be made available by the authors, without undue reservation.

## Ethics Statement

The studies involving human participants were reviewed and approved by Ethics Committee for Clinical Trials of Venice Province and IRCCS San Camillo Hospital. The patients/participants provided their written informed consent to participate in this study.

## Author Contributions

MM, DM, and GF designed the study. MM and GF acquired and processed the data and wrote the manuscript. LC-G provided technical support. All authors reviewed and approved the manuscript.

## Conflict of Interest

The authors declare that the research was conducted in the absence of any commercial or financial relationships that could be construed as a potential conflict of interest.

## Publisher’s Note

All claims expressed in this article are solely those of the authors and do not necessarily represent those of their affiliated organizations, or those of the publisher, the editors and the reviewers. Any product that may be evaluated in this article, or claim that may be made by its manufacturer, is not guaranteed or endorsed by the publisher.

## Abbreviations

LF: low frequency
HF: high frequency
CJV: coefficient of joint variation.
